# Immune System Involvement in the Pathogenesis of JC Virus Induced PML: What is Learned from Studies of Patients with Underlying Diseases and Therapies as Risk Factors

**DOI:** 10.3389/fimmu.2015.00159

**Published:** 2015-04-28

**Authors:** Maria Chiara G. Monaco, Eugene O. Major

**Affiliations:** ^1^Laboratory of Molecular Medicine and Neuroscience, National Institute of Neurological Disorders and Stroke, National Institutes of Health, Bethesda, MD, USA

**Keywords:** progressive multifocal leukoencephalopathy, JCV, immunomodulatory therapy, T cell immune response, multiple sclerosis, natalizumab

## Abstract

The human polyomavirus JC PyV lytic infection of oligodendrocytes in the human brain results in the demyelinating disease progressive multifocal leukoencephalopathy, PML. JCV is a common virus infection in the population that leads to PML in patients with underlying diseases and therapies that cause immune deficiencies or modulate immune system functions. Patients may have high levels of antibody to JCV that neither protect them from PML nor clear the infection once PML is established. Cell-mediated immunity plays a more effective role in clearing initial or reactivated JCV infection before PML occurs. However, patients with underlying diseases and therapies for treatment are at high risk for PML. MS patients on natalizumab are one of the categories with the highest incidence of PML. Natalizumab is a humanized monoclonal antibody targeting α4 integrins that prevents inflammatory cells from entering the brain and it has been used as a treatment for MS. A number of studies have investigated the occurrence of PML in these patients and their cell-mediated immune profile that might gain insight into the mechanism that ties natalizumab with a high risk of developing PML. It seems that cells of the immune system participate in the pathogenesis of PML as well as clearance of JCV infection.

## Introduction

During the course of viral infections, cells of the immune system function to protect the host by clearing virus and infected cells from the peripheral circulation in blood and numerable tissue compartments. The principle immune system compartments include thymus, bone marrow, lymph nodes, spleen, and tonsils. The immune cells within these compartments are predominantly precursor and mature T and B lymphocytes as well as multipotential stem cells of the hematopoietic system found in bone marrow. While immune system cells in human infection with JC virus do play a critical role in clearing JCV, these cells also participate in the pathogenesis of infection leading to PML. Evidence of JCV infection in mononuclear cells in spleen and bone marrow was first seen in a PML/AIDS patient and another patient with no recognizable immune deficiency (1). B lymphocytes infected with JCV were found in the bone marrow specimens and those cells were implicated as carriers of virus from the periphery to the brain after investigation of cerebral biopsy samples. A number of other reports of JCV infection in peripheral lymphocytes and bone marrow have further linked these cells with viral latency in compartments from which reactivation from latency can occur in the setting of an immune compromised state (2). Investigation of peripheral blood mononuclear cells and bone marrow in HIV-1 infected and non-infected individuals detailed the presence of JCV DNA and T-antigen in bone marrow samples in both PML and non-PML patients, highlighting the importance of bone marrow as a viral reservoir (3). In another study of hematopoietic stem cell transplant (HSCT) patients, a number of patients were viremic before transplant as measured in blood and urine samples that did not diminish for approximately 1 year after transplant. While these patients had anti-JCV antibody, their humoral antibody response did not lower the presence of virus. Cell-mediated responses of both CD4 and CD8 did develop over time, however, but that occurred over 1 year (4). These studies further implicate bone marrow and immune system cells in the pathogenesis of viral induced PML.

## JCV in Cell Compartments

Since there is a high risk of PML in MS patients treated with natalizumab, a humanized monoclonal antibody against α_4_β_1,7_ integrin, a cohort of patients were tested for JCV DNA in peripheral blood (5). Blood was collected from patients before the first dose and during the initial dosing of drug for up to 10 months. Peripheral mononuclear cells were identified and sorted into CD34^+^, CD19^+^, and CD3^+^ cells. After several weeks of treatment, the level of CD34^+^ cells in the periphery was substantially higher than normal, attributed to natalizumab effects on migration of cells out of bone marrow, as previously reported (6). Another cohort of MS patients who were treated with natalizumab for over 24 months had one blood sample collected for viral analysis. None of the MS patients from either cohort had PML. The results showed that viral DNA could be found in 50% of patients, some at baseline, in at least one compartment of either CD34^+^ or CD19^+^ cells mostly at 3 and 6 months. Viral DNA was never detected in CD3^+^ population of T cells that acted as controls. In the cohort receiving more than 24 doses of natalizumab, 44% showed viral DNA in at least one compartment. There was a higher prevalence of viral DNA in CD34^+^ cells than CD19^+^ cells from both groups. Some of these patients tested negative for antibodies to JCV although both CD4^+^ and CD8^+^ responses were present indicating the potential for lack of antigen presentation to mount a measureable antibody response (7). The direct observation of intracellular viremia in CD34^+^ and CD19^+^ cells suggests that these cells can harbor JCV.

Based upon the identification of JCV infection in bone marrow and B lymphocytes, a possible mechanistic link between natalizumab and PML was described in 2009 (8) when many new cases of PML were being diagnosed. This was several years after the first index cases and following the FDA release of natalizumab back to the market. The occurrence of PML in MS cases treated with natalizumab continues to accrue at a rate of approximately 10 new cases per month. Several factors may account for the high incidence of PML in these patients and not in MS patients not treated with natalizumab. One of the unique effects of natalizumab is its forced migration of CD34^+^ cells from the bone marrow to the peripheral circulation. Since JCV may remain persistent in these cells, it is not surprising that at some time; latently infected CD34^+^ cells find their way into the blood with the ability to differentiate to a B lymphocyte lineage (9). An illustration of the mechanism of JCV pathogenesis in PML in association with natalizumab is reported in the perspective article on the New England Journal of Medicine (8). A gene array analysis of blood cells showed that a number of genes were upregulated including genes whose function is B cell differentiation in the POU domain family (10). One of these genes is for the transcription factor, Spi B, which was shown to bind to the regulatory region of JCV (10–13). A recent study also showed that natalizumab regulates miRNA-126 that controls some of these genes in a temporal dependent manner (14). From initiation of treatment to 24 months, natalizumab down regulates miRNA-126 that modulates some immune cell functional pathways but upregulates genes after 24 months. Spi B is affected by this modulation that may account for the low incidence of PML in MS treated natalizumab patients up to 24 months after which PML occurs at its highest rate, now seen in 1:80 of these patients. This rate equals or exceeds the rate in HIV-1 infected patients in which PML remains an AIDS defining illness.

**Figure 1:**
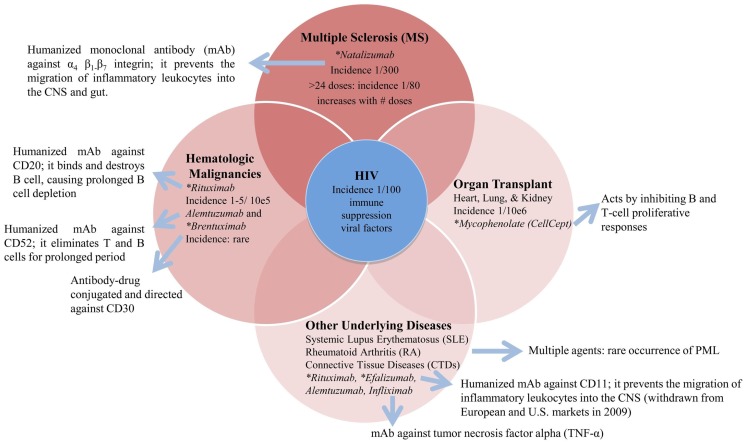
**Underlying diseases and associated therapies with a risk for PML**. *FDA black box warning for PML risk (http://www.fda.gov/safety/medwatch).

There are other therapies and underlying diseases that are associated with the occurrence of PML such as hematologic malignancies, rheumatic diseases such as rheumatoid arthritis, systemic lupus erythematosus, and organ transplants. In these patients, the incidence of PML is several orders of magnitude less than either AIDS patients or MS patients treated with natalizumab as shown in the Figure 1.

A new anti-adhesion biologic, vedolizumab, used to treat inflammatory bowel disorders, shows no evidence of PML as reported in two separated phase 3, placebo-controlled studies (15, 16). This new monoclonal antibody is specific to the gut integrin, and it blocks the entire α_4_β_7_ heterodimers, after binding the MAdCAM-1 ligand, as oppose to only the recognition of the α_4_ subunit (as natalizumab through the VCAM-1 ligand), and therefore does not affect the α_4_β_1_-mediated leukocytes transmigration into the nervous system. Whether JCV infects gut tissue has been investigated. There was a report presenting data comparing JCV seroreactivity in patients with colorectal cancer (CRC) or adenomas (17). This study was performed on a small number of patients and did not report a correlation between the expression of JCV T-antigen in the colon tissues of those patients and JCV seroreactivity and therefore leaves the question of JCV infection as a cause of CRC unanswered (18, 19). There have been no directed studies of mucosal (gut) mediated immunity to JCV infection as yet.

## PML Risk Assessments

The first two cases of PML in MS patients treated with natalizumab occurred almost 10 years ago. Since then a significant focus has been on the assessment of risk factors that would assist neurologists and patients to make decisions on the use of particularly biologics, like natalizumab, which modulate the immune system. The measurement of the risk of PML associated with natalizumab treatment is currently based on three factors: presence of anti-JC virus antibodies or serologic status, previous treatment using immunosuppressants prior to natalizumab, and the duration of natalizumab treatment (20–23). The increased duration of natalizumab treatment and serologic status are two consistent risk factors for the possible development of PML. Currently, the serologic status of the patients seems to leave room for more investigations as more patients are being treated with natalizumab. Data from several laboratories indicate that JCV serostatus does not seem to detect some patients who had been infected with JCV. Consequently, a seronegative JCV results may not be associated with prior JCV infection. Berger et al. (24) reported a higher frequency, over 30%, of false negative results for JCV antibody than what previously described, as 2–3% (25, 26). Another study (27) identified patients with MS who did not have PML and did not have antibody responses to JCV even during active viral infection as measured by viremia and viruria. Although serologic status is an effective measurement for exposure to JCV, it may be not sufficient to evaluate the complete risk of PML during the course of natalizumab treatment. A recent paper (28) investigated the use of a second generation ELISA test that proposes to further differentiate PML risk. This study showed that, in natalizumab-treated patients with JCV positive serostatus and no prior immunosuppressants, it is feasible to further cluster those patients into lower and higher PML risk groups based on serum or plasma anti-JCV antibodies level, measured as JCV-index (28). The correlation of serostatus with other factors remains an open question in particular in the cases of negative serological data where both JCV viremia and cell-mediated immune responses to JCV were detected (7, 27). This observation suggests that JCV is dynamically replicating and disseminating from sites of latency or low-grade persistent infection prior to the development of PML. Moreover, considering that the majority of PML patients house JCV-specific antibodies before or at the start of the disease, the humoral immune response may not be adequate to protect the host from JCV infection leading to PML (26). Therefore, JCV-specific cellular immune responses have been investigated.

## PML and Immune System

Such technical advances have resulted in new knowledge on JCV, PML, and immune system interactions. One example is the development of chimeric immune receptors (CIRs) to create T cells genetically altered, called “designer T cells” that target the elected antigen specificity (29). In that work, the authors reported that specific tetramers were able to identify target cells presenting JCV antigens and to perform activation and signaling with cytokine production as well as cytotoxic activity (29). CD8^+^ cytotoxic T cells effect the JCV-specific cellular immune responses have been more thoroughly investigated (30, 31). To support further detailed studies on JCV-specific CTL response, viral epitope peptides have been characterized. Those peptides are presented to CTLs by the expressed MHC class I molecule HLA-A*0201 (30). The authors assembled a tetramer reagent and used that complex as a tool to investigate the role of JCV-specific CTL in the immunopathogenesis of PML. JCV-specific CD8^+^ T cells were measurable in 91% of PML survivors, while they were not detectable in any of the PML progressors (30). This study suggests that the detection of JCV-specific CTL in PBMC of individuals with PML may be a good prognostic marker of the evolution and progression of the disease and could be essential for the prevention and the resolution of PML. Other studies, instead, focused on the important role of JCV-specific CD4^+^ T cells in the control of JCV infection based on the knowledge of the increased risk of PML in AIDS patients with low levels of CD4^+^ lymphocytes, and in patients with idiopathic CD4^+^ lymphocytopenia (32). A recent study (33) reported the presence of JCV-specific CD4^+^ T cells in the brain of PML patients undergoing PML–IRIS syndrome (immune reconstitution inflammatory syndrome). IRIS is a consequence of the replacement of CD4^+^ cells in AIDS patients or of the restoration of the trafficking of immune system cells following plasma exchange (PLEX) to accelerate the removal of natalizumab in MS patients. The authors emphasize the role of CD4^+^ T cells responses and conclude that PML–IRIS and the consequent inflammation are life threatening events. However, the presence of specific T cells to JCV plays a dominant role in controlling JCV infection.

It is assumed that such cell mediated responses to JCV clears infection and therefore should reduce the risk of PML in high risk patients (32).

Furthermore, recent studies (7, 34, 35) showed that both CD4^+^ and CD8^+^ T cell responses were identified against not only the JCV-VP1, the major capsid protein (34, 35), but also other viral proteins (7) as measured *ex vivo* in the peripheral blood of healthy individuals and in natalizumab-treated patients. Other longitudinal studies reported highly predominant anti-JCV memory and effector T cell response in natalizumab-treated MS patients (36–38). Those results measured JCV-specific T cell responses directed against the VP1 protein by interferon gamma production in viral peptide stimulated T cells. Data from Perkins et al. (7) demonstrated that immune control of JCV infection might depend on both CD4 and CD8 T cell responses directed at most of the virus proteins assessing the quality of the immune responses by cytokine release assays. In those studies, it was also shown that patients who developed PML under natalizumab treatment lacked adequate CD4 T cells responses and had upregulated Il-10 production. These data suggest that monitoring of both humoral and cellular immune responses might better assess risk of PML in natalizumab-treated patients. The chronological investigations on humoral and T cell-mediated responses are listed in Table 1.

**Table 1:** **Assessment of immune responses to JCV infection**.

	Assay	Reference
**HUMORAL IMMUNE RESPONSE**		
JCV capability to agglutinate type O erythrocytes	Hemagglutination inhibition assay (HIA)	(39)
Recombinant VP1^a^ produces virus-like particles (VLPs)	Enzyme-linked immunosorbent assay (ELISA)	(40, 41)
Recombinant VP1^a^ produces virus-like particles (VLPs)	ELISA	(42)
Recombinant VP1^a^ produces virus-like particles (VLPs)	ELISA	(43)
Two-step assay to detect anti-JCV antibodies using VLPs	ELISA	(25)
**CELLULAR IMMUNE RESPONSE**		
JCV-specific CD8^+^ T cell responses	Cytotoxic T lymphocyte assay (CTL)	(30, 44)
Tetramer with anti-JCV TCR (T cell receptor) exhibits T cell activation	Chimeric immune receptors (CIRs)	(29)
CD4 and CD8-T cell responses specific to VP1 protein	ELISpot and intracellular cytokine staining (ICS) (IFN-γ)	(34)
CD4 and CD8-T cell responses specific to all JCV proteins	ICS (IFN-γ; TNF-α, IL-2; IL-10)	(7)
CD4 T cell responses specific to all JCV proteins	ICS (IFN-γ)	(33)

*^a^JC virus, major capsid protein, VP1*.

## Conflict of Interest Statement

The authors declare that the research was conducted in the absence of any commercial or financial relationships that could be construed as a potential conflict of interest.
